# Influential Periods in Longitudinal Clinical Cardiovascular Health Scores

**DOI:** 10.1093/aje/kwab149

**Published:** 2021-05-20

**Authors:** Amy E Krefman, Darwin Labarthe, Philip Greenland, Lindsay Pool, Liliana Aguayo, Markus Juonala, Mika Kähönen, Terho Lehtimäki, R Sue Day, Lydia Bazzano, Vito M R Muggeo, Linda Van Horn, Lei Liu, Larry S Webber, Katja Pahkala, Tomi T Laitinen, Olli Raitakari, Donald M Lloyd-Jones, Norrina B Allen

**Keywords:** adolescence, cardiovascular epidemiology, cardiovascular health, cohort studies, longitudinal studies, prevention, risk factors

## Abstract

The prevalence of ideal cardiovascular health (CVH) among adults in the United States is low and decreases with age. Our objective was to identify specific age windows when the loss of CVH accelerates, to ascertain preventive opportunities for intervention. Data were pooled from 5 longitudinal cohorts (Project Heartbeat!, Cardiovascular Risk in Young Finns Study, The Bogalusa Heart Study, Coronary Artery Risk Development in Young Adults, Special Turku Coronary Risk Factor Intervention Project) from the United States and Finland from 1973 to 2012. Individuals with clinical CVH factors (i.e., body mass index, blood pressure, cholesterol, blood glucose) measured from ages 8 to 55 years were included. These factors were categorized and summed into a clinical CVH score ranging from 0 (worst) to 8 (best). Adjusted, segmented, linear mixed models were used to estimate the change in CVH over time. Among the 18,343 participants, 9,461 (52%) were female and 12,346 (67%) were White. The baseline mean (standard deviation) clinical CVH score was 6.9 (1.2) at an average age of 17.6 (8.1) years. Two inflection points were estimated: at 16.9 years (95% confidence interval: 16.4, 17.4) and at 37.2 years (95% confidence interval: 32.4, 41.9). Late adolescence and early middle age appear to be influential periods during which the loss of CVH accelerates.

## Abbreviations

BMI: body mass index
BP: blood pressure
CARDIA: Coronary Artery Risk Development in Young Adults
CVH: cardiovascular health
STRIP: Special Turku Coronary Risk Factor Intervention Project
Young Finns: Cardiovascular Risk in Young Finns Study

Heart disease remains the leading cause of death in the United States, and the current prevalence of ideal cardiovascular health (CVH) among adults in the United States is less than 5% (1, 2). Ideal CVH is a concept, defined by the American Heart Association, and includes 4 health factors (body mass index (BMI), blood pressure (BP), total cholesterol, and fasting blood glucose) and 3 health behaviors (smoking, physical activity, and diet) (2). Ideal CVH has been linked to a broad range of improved health outcomes, including greater longevity and quality of life, and lower risk for heart disease, incident cancer, and dementia (3). Ideal CVH prevalence decreases with age, but whether declines are consistent across the life course is unknown. Identifying if there are ages when the rate of CVH decline accelerates could facilitate better targeting of preventive efforts to these periods and understanding of the causes and drivers of loss of CVH, which may be due to specific developmental periods across the life course.

Recent advances in estimation of change points allow us to estimate when decline in CVH begins to accelerate across the life course. Previous studies have looked at overall patterns or trajectories of CVH over time to determine how the trajectories correspond to future risk and characterize which children may be more likely to be in specific risk categories; these studies have provided important information about risk stratification (4, 5). The present study expands on this concept by decomposing risk over time to determine specific periods or ages, which has not been quantitatively assessed in previous studies.

In this study, to identify opportunities to promote and maintain ideal CVH throughout the life course, we sought to estimate influential age windows when the rate of decline in CVH changes at the population level. In addition to identifying these influential change points, we wanted to determine whether there are sex differences in the rate of change between change points, as well as how the individual metrics changed over time. We hypothesized CVH declines are not consistent across the life course and there is at least 1 age when the rate of decline significantly changes.

## METHODS

### Cohorts and participants

This study included 18,343 individuals from 5 cohorts: Project HeartBeat! (6), Cardiovascular Risk in Young Finns Study (Young Finns) (7), The Bogalusa Heart Study (8), Coronary Artery Risk Development in Young Adults (CARDIA) (9), Special Turku Coronary Risk Factor Intervention Project (STRIP) (10). Details of harmonizing these cohorts’ data are described elsewhere (4). In brief, clinical measurements were collected for each cohort at in-person examinations and questionnaires were used to collect demographic and behavioral data over varied ages and lengths of time. By including cohorts that spanned overlapping age windows, we could cover a wider range, from ages 8 through 55 years. Participants were included in the analysis if they had at least 1 clinical CVH score (all 4 clinical CVH components measured at the same examination), as described the next section, CVH factors (Web Figure 1) (available at https://doi.org/10.1093/aje/kwab149). Parental education data, categorized as years of education, were collected as a proxy for socioeconomic status.

### CVH factors

CVH factors available from all studies included BMI, BP, and lipid and fasting glucose levels. BMI was calculated from measured weight (in kilograms) and the square of the height (in meters). Before calculating the CVH score, BMI was converted to an age- and sex-specific percentile for those younger than 20 years, using the Centers for Disease Control and Prevention calculation (11).

Systolic and diastolic BP were measured in all cohorts at every examination. BP was converted to percentile values for those younger than 18 years, using the pediatric hypertension guidelines published in 2017 (12). Levels of fasting serum lipids and fasting blood (plasma or serum) glucose were measured at multiple examinations for each cohort.

Each BMI, BP, fasting glucose, and total cholesterol observation was considered ideal, intermediate, or poor, on the basis of American Heart Association criteria (Table 1) (2, 4). To create a CVH score, points were assigned for the levels of each factor: 0 for poor, 1 for intermediate, and 2 for ideal. Points from the 4 CVH factors measured at the same examination were summed to create a clinical CVH score. This score ranged from 0 to 8, with higher CVH score indicating better CVH. This score is associated with the future risk of heart disease: maintaining a higher score is associated with less risk (4).

**Table 1:** Clinical Cardiovascular Health Score Components, Cardiovascular Health Pooled Cohort, 1973–2012

	**Body Mass Index** ^**a**^		**Total Cholesterol, mg/dL**		**Blood Pressure, mm Hg**		**Fasting Blood Glucose, mg/dL**	
**Health Category**	**Age ≥20 Years**	**Age <20 Years, percentile**	**Age ≥20 Years**	**Age <20 Years**	**Age ≥18 Years**	**Age <18 Years, percentile**	**Age ≥20 Years**	**Age <20 Years**
Ideal	<25.0	<85th	<185, unmedicated	<185, unmedicated	<120/80, unmedicated	<90th	<100, unmedicated	<100, unmedicated
Intermediate	25.0–29.99	85th–95th	185–219 or treated to < 185	185–219 or treated to <185	SBP 120–139 or DBP 80–89, or treated to <120/80	90th–95th or SBP ≥120 or DBP ≥80	100–125 or treated to < 100	100–125 or treated to < 100
Poor	≥30.0	>95th	≥220	≥220	SBP ≥140 or DBP ≥90	>95th	≥126	≥126

Abbreviations: DBP, diastolic blood pressure; SBP, systolic blood pressure.

^a^ Weight (kg)/height (m)^2^

### CVH behaviors

CVH behaviors (i.e., smoking, diet, and physical activity) were not measured consistently across follow-up and thus were not included in the clinical CVH score. However, baseline behaviors were captured in each cohort and were categorized as ideal or not ideal on the basis of the American Heart Association criteria (Web Table 1) to describe the characteristics of the sample in Table 2 (2, 13).

**Table 2:** Demographic Characteristics, Clinical Measures, and Covariates at Each Individual’s First Examination, by Sex, Cardiovascular Health Pooled Cohort,^a^ 1973–2012

**Characteristic**	**Male (*n* = 8,882)**			**Female (*n* = 9,461)**		
	**Mean (SD)**	**No.**	**%**	**Mean (SD)**	**No.**	**%**
White race		6,058	68.2		6,288	66.5
Age, years	17.4 (8.1)			17.8 (8.1)		
Cohort						
Young Finns		1,486	16.7		1,650	17.4
Project HeartBeat!		201	2.3		208	2.2
CARDIA		2,315	26.1		2,783	29.4
Bogalusa Heart Study		4,628	52.1		4,582	48.4
STRIP		252	2.8		238	2.5
Clinical measures						
BMI^b^	21.3 (4.9)			21.5 (5.5)		
BMI %, by age and sex^c^	52.8 (29.9)			53.5 (30.1)		
SBP, mm Hg	109.5 (13.1)			105.9 (11.1)		
SBP %^d^	47.7 (27.0)			50.2 (26.8)		
DBP, mm Hg	59.1 (15.1)			59.4 (13.0)		
DBP %^d^	24.9 (22.4)			28.9 (24.1)		
Total cholesterol, mg/dL	169.8 (34.0)			173.2 (33.1)		
Fasting glucose, mg/dL	85.9 (12.7)			83.0 (14.4)		
Ideal behavior score^e^						
Smoking		4,922	62.4		5,541	64.8
Diet		1,088	23.6		1,793	32.6
Physical activity		1,781	38.3		1,179	22.4
Maternal education^f^						
≤6 years		231	3.6		313	4.3
7–9 years		971	14.9		1,131	15.5
10–12 years		2,869	44.1		3,164	43.4
13–16 years		1,947	29.9		2,176	29.8
>16 years (graduate school)		483	7.4		508	7.0
Paternal education, years^f^						
≤6		270	4.7		361	5.7
7–9		1,045	18.3		1,215	19.0
10–12		2,348	41.1		2,574	40.3
13–16		1,609	28.1		1,719	26.9
>16 (graduate school)		447	7.8		514	8.1
Clinical CVH score	6.8 (1.3)			7.0 (1.2)		

Abbreviations: BMI, body mass index; CARDIA, Coronary Artery Risk Development in Young Adults; CVH, cardiovascular health; DBP, diastolic blood pressure; SBP, systolic blood pressure; SD, standard deviation; STRIP, Special Turku Coronary Risk Factor Intervention Project, Young Finns, Cardiovascular Risk in Young Finns Study

^a^ The CVH pooled cohort comprised participants in the Bogalusa Heart Study, CARDIA, Project HeartBeat!, STRIP, and Young Finns.

^b^ Weight (kg)/height (m)^2^.

^c^ The BMI% calculation was only available for those who were < 20 years old at baseline. In analytical sample BMI%, *n* = 11,777;

^d^ SBP%, and DBP% data were only available for those who were < 18 years old at baseline. In the analytic sample BP%, *n* = 10,627.

^e^ Behavior scores were not captured at the first visit for every participant; these scores represent their first available score for that behavior.

^f^ Parent education data were not available for all individuals.

### Statistical analysis

Demographic variables, CVH factors, CVH behaviors, and parental education measured at each participant’s baseline examination were described and stratified by sex (Table 2) and by age (Web Table 2). We estimated the age at which distinct change points occurred in the mean clinical CVH score from age 8 years through age 55 years, using piecewise linear regression extended to a longitudinal framework in the unpublished R (R Foundation for Statistical Computing, Vienna, Austria) function “segmented.lme” (14). This approach allows for the estimation of interpretable parameters such as slopes and change points.

First, the mixed model was fit with a random participant intercept and slope and adjusted for race, sex, and cohort using “nlme” in R (15). Next, we implemented an iterative procedure to estimate the change point(s) of the mixed model, using “segmented.lme.” The change point(s) and their asymptotic 95% confidence intervals were estimated using a maximum likelihood approach. After the change point(s) were estimated, linear, quadratic, and cubic mixed models, and segmented linear mixed models with 1 and 2 change points were compared. The best fit was determined using Akaike Information Criteria, Bayesian Information Criteria, and likelihood ratio tests (Web Table 3). Because there was a significant interaction among age, race, and sex (*P* < 0.001) in the initial mixed model, analyses were stratified by sex, and models were fit using the same procedure.

After estimating the change point(s), we tested the difference between the slopes in each age window (8.0–16.9 years, 17.0–36.9 years, and 37.0–55.99 years) by sex. The change in CVH score over time was modeled as a multivariable, piecewise linear regression model with 2 knots (determined by the aforementioned change point estimates), adjusting for race and cohort. Interaction terms between age and sex were included in the model to test for sex differences in the magnitude of change in CVH over time.

To further test the robustness of our models, we calculated means at ages 8, 17, 37, and 55 years for each component of the CVH score separately (i.e., BMI, BP, and fasting glucose and total cholesterol levels) using their original continuous values (e.g., continuous systolic BP in millimeters of mercury rather than poor, intermediate, or ideal BP). Although age- and sex-specific percentiles were used to score BMI and BP into ideal categories for persons younger than 20 years (for BMI) and 18 years (for BP), continuous metrics were used to calculate means for the purposes of continuity across the full age range. We used *t* tests to determine if there was a significant difference between the means for males and females at each age.

Analyses were performed using SAS, version 9.4 (SAS Institute Inc, Cary, North Carolina) and R, version 3.3.3 (16) using the nlme package (15) and an extension of the segmented package written by Muggeo et al (14, 17) for use with linear mixed models. Statistical significance was set a priori at *P* < 0.05.

## RESULTS

Among the 18,343 participants in our sample, 9,461 (52%) were female and 12,346 (67%) were White. At the baseline examination, 10,463 (64%) had ideal smoking status, whereas only 16% had ideal diet or ideal physical activity scores. Mean age at baseline was 17.6 (standard deviation, 8.1) years. The mean clinical CVH score at baseline was 6.9 (standard deviation, 1.2) out of a possible 8.0 (Table 2).

**Figure 1:**
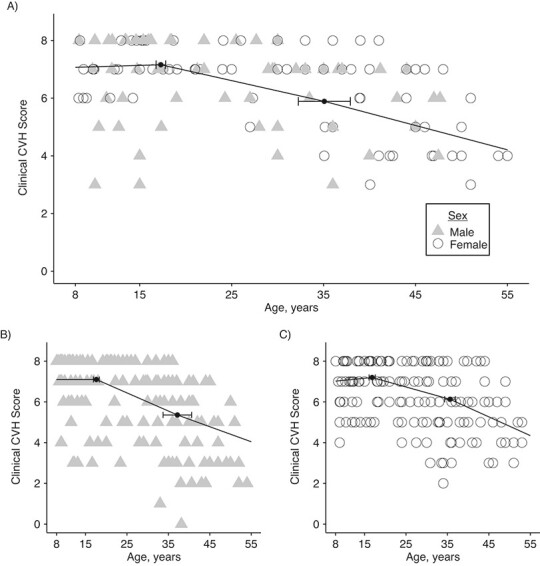
Plots of unadjusted segmented mixed models (fixed effects only), cardiovascular health (CVH) pooled cohort, 1973–2012. A) Overall model (change points: 17.3 and 35.1; 95% confidence intervals (CIs): 16.8, 17.8 and 32.3, 37.9, respectively). Stratified models: B) males (change points: 17.1 and 37.2; 95% CIs: 16.9, 18.3 and 33.7, 40.6, respectively) and C) females (change points: 16.8 and 35.6; 95% CIs: 16.1, 17.6 and 34.3, 36.9, respectively). (For illustrative purposes, data points from 50 randomly selected participants are shown).

**Figure 2:**
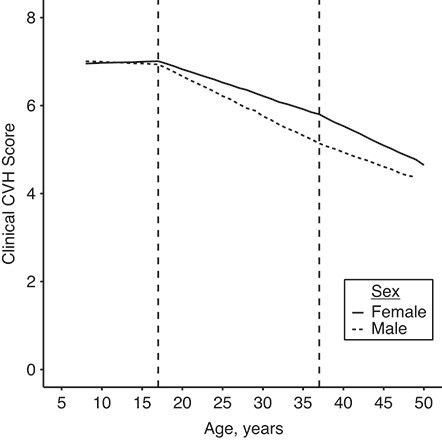
Adjusted segmented mixed model, by sex, cardiovascular health (CVH) pooled cohort, 1973–2012. The reference group, Black males and females from Bogalusa Heart Study (1973–2010), is shown here. Dashed vertical lines indicate the knots at 17 and 37 years. Clinical CVH score ranges from 0 to 8, with 8 being the most ideal.)

In modeling clinical CVH scores over time, the adjusted, segmented, linear mixed model with 2 change points and random intercept and slope provided the best fit to the data compared with other models (Web Table 3). Change points in unadjusted models were estimated at 17.3 and 35.1 years (95% confidence intervals (CI): 16.8, 17.8; and 32.3, 37.9, respectively), as shown in Figure 1A. The adjusted model had change points at 16.9 (95% CI: 16.4, 17.4) and 37.2 (95% CI: 32.5, 41.9) years. This second inflection point’s uncertainty, causing a somewhat wider confidence interval, reflected a much smaller acceleration in the rate of decline. Before the first change point in both the unadjusted and adjusted models, beginning at age 8 years in the present data, the rate of decline was close to 0 (0.01 points/year; 95% CI: −0.004, 0.02 in the adjusted model). After the first change point, the rate of decline significantly increased to −0.07 points/year (95% CI: −0.08, −0.07). At that rate, an individual would lose 1.5 points in their clinical CVH score in the 20-year span between ages 17 and 37 years. After age 37 years, the rate of decline increased further to −0.08 (95% CI: −0.09, −0.08) points/year (Figure 2).

The stratified models determining change points for males and females did not differ significantly from each other or the overall estimates (male, unadjusted (Figure 1B); male, adjusted: 16.93 (95% CI: 16.25, 17.61) and 35.69 (95% CI: 33.09, 38.29); female, unadjusted (Figure 1C); female, adjusted: 16.71 (95% CI: 15.94, 17.47) and 36.06 (95% CI: 34.62, 37.49)). However, the slopes of the various segments by sex were significantly different in all 3 age windows: 8 to < 17 years, 17 to < 37 years, and 37 through 55 years (Table 3). Before age 17 years, female CVH held steady while male CVH decreased 0.01 points/year (95% CI: −0.02, −0.01; for difference between sexes, *P* = 0.001). Between ages 17 and 37 years, CVH declined at a faster rate for men than for women (−0.09 vs. −0.06; *P* < 0.001). After age 37 years, CVH declined more rapidly for women than men (−0.09 vs. −0.07; *P* < 0.001), which suggests a different pattern of change by sex. Models stratified by sex and race were not substantively different from the models stratified by sex only (Web Table 4 and Web Figure 2).

**Table 3:** Mixed Model Parameter Estimates of Mean Clinical Cardiovascular Health Score^a^ Over Time, by Sex, Cardiovascular Health Pooled Cohort,^b^ 1973–2012

**CVH Outcome**	**Unadjusted**				**Adjusted** ^**c**^			
	**Mean**	**95% CI**	***P* Value**	**Total Δ Over Segment**	**Mean**	**95% CI**	***P* Value**	**Total Δ Over Segment**
Mean CVH score for 8-year-old children								
Female (referent)	7.09	7.05, 7.13	<.001		6.95	6.89, 7.00	<.001	
Male	7.15	7.11, 7.19	<.001		7.00	6.94, 7.06	<.001	
Difference in mean CVH score for 8-year-old children between sexes	0.06	0.00, 0.11	0.04		0.05	0.00, 0.11	0.06	
Segment-specific slope, Δ/year								
Age group, years, female								
8.0–16.9	0.00	−0.01, 0.01	0.83	0.01	0.00	0.00, 0.01	0.38	0.01
17.0–36.9	−0.06	−0.06, −0.06	<.001	−1.21	−0.06	−0.06, −0.06	<.001	−1.2
37.0–55.9	−0.09	−0.09, −0.09	<.001	−1.63	−0.09	−0.09, −0.09	<.001	−1.63
Age group, years, male								
8.0–16.9	−0.01	−0.02, −0.01	<.001	−0.12	−0.01	−0.02, 0.00	<.001	−0.12
17.0–36.9	−0.09	−0.09, −0.09	<.001	−1.8	−0.09	−0.09, −0.09	<.001	−1.79
37.0–55.9	−0.07	−0.07, −0.06	<.001	−1.24	−0.07	−0.07, −0.06	<.001	−1.25
Difference in segment-specific slope between sexes, by age group, years								
8.0–16.9: male − female	−0.01	−0.02, −0.01	0.001	−0.13	−0.01	−0.02, −0.01	<.001	−0.13
17.0–36.9: male − female	−0.03	−0.03, −0.03	<.001	−0.59	−0.03	−0.03, −0.03	<.001	−0.59
37.0–55.9: male − female	0.02	0.02, 0.03	<.001	0.38	0.02	0.01, 0.03	<.001	0.38

Abbreviations: CVH, cardiovascular health; Δ, change in, or difference.

^a^ Clinical CVH score ranges from 0 to 8, with 8 being the most ideal.

^b^ CVH pooled cohort comprised participants in the Bogalusa Heart Study, Coronary Artery Risk Development in Young Adults, Project HeartBeat!, Special Turku Coronary Risk Factor Intervention Project, and Cardiovascular Risk in Young Finns Study.

^c^ Adjusted for age at baseline, race, and cohort. Numbers presented are for Black individuals from the Bogalusa Heart Study (Bogalusa, Louisiana).

The sex-specific means at each influential age (i.e., 8, 17, 37, and 55 years) help explain the differences in the change in CVH score over time (Table 4 and Web Figure 3). Behavioral metrics, diet, physical activity, and smoking data were collected less frequently than were the clinical metrics; mean scores (poor (0) to ideal (2)) are shown in Web Table 5.

**Table 4:** Mean Values of Each Clinical Metric at the Change Points, by Sex, CVH Pooled Cohort,^a^ 1973 to 2012

	**Age Group**											
	**8-Year-Olds**						**17-Year-Olds**					
**Clinical Metric**	**Female**		**Male**		**Difference in Mean (μ**_**Male**_ **− μ**_**Female**_ **)**		**Female**		**Male**		**Difference in Mean (μ**_**Male**_ **− μ**_**Female**_ **)**	
	**Mean**	**SD**	**Mean**	**SD**	**Difference**	**95%CI**	**Mean**	**SD**	**Mean**	**SD**	**Difference**	**95%CI**
BMI^b^	17.2	3.1	17.0	2.9	−0.2	−0.5 to 0.1	22.2	4.5	22.3	4.1	0.1	−0.3 to 0.5
Systolic BP, mm Hg	96.5	8.8	96.8	8.3	0.3	−0.6 to 1.2	110.7	9.0	117	11.7	6.3	5.2 to 7.4^c^
Diastolic BP, mm Hg	45.1	12.0	44.1	10.9	−1.0	−2.1 to 0.2	61.1	8.6	59.6	10.3	−1.5	−2.4 to −0.5 ^c^
Total cholesterol, mg/dL	167.6	27.9	164.2	25.8	−3.4	−6.1 to −0.6^c^	163.3	30.6	151.1	28.2	−12.2	−15.2 to −9.2 ^c^
Fasting blood glucose, mg/dL	80.7	8.0	82.4	9.0	1.8	0.9 to 2.6^c^	81.6	8.2	86	8.4	4.4	3.5 to 5.2^c^
	**37-Year-Olds**						**55-Year-Olds**					
BMI^b^	27.5	7.2	27.6	5.4	0.1	−0.5 to 0.7	31.4	7.7	30.3	6.1	−1.1	−2.6 to 0.4
Systolic BP, mm Hg	109.7	14.4	116.2	12.9	6.5	5.1 to 7.9^c^	117.6	15.9	121.4	15.9	3.7	0.2 to 7.3^c^
Diastolic BP, mm Hg	70.7	11	75	10.4	4.3	3.2 to 5.4^c^	72.6	11.2	73.9	10.3	1.3	−1.1 to 3.7
Total cholesterol, mg/dL	181.4	33.1	193.3	40.5	11.9	8.1 to 15.7^c^	201.7	36.3	182.4	35.9	−19.3	−27.4 to −11.2^c^
Fasting blood glucose, mg/dL	90.1	18	96.3	25.9	6.1	3.8 to 8.5^c^	95.9	15.8	104	21.9	8	3.6 to 12.4^c^

Abbreviations: BMI, body mass index; BP, blood pressure; CI, confidence interval; SD, standard deviation.

^a^ CVH pooled cohort comprised participants in the Bogalusa Heart Study, Coronary Artery Risk Development in Young Adults, Project HeartBeat!, Special Turku Coronary Risk Factor Intervention Project, and Cardiovascular Risk in Young Finns Study.

^b^ Weight (kg)/height (m)^2^.

^c^ *P* < 0.05 for *t* test.

BMI increased steadily for both males and females over time; however, the difference between the 2 sexes was not significant at any of the 4 ages. The change in BMI over time was consistent with the change in overall score over time in that it remained at ideal levels at ages 8 and 17 years, declined to intermediate by age 37 years, and to poor by age 55 years. BP started out similarly for males (97/44 mm Hg) and females (97/45 mm Hg). Although mean systolic and diastolic BP both increased for each sex, they remained at ideal levels through age 55 years. Males had a steeper increase in systolic BP than females before age 17 years (+6.0 mm Hg), but after holding relatively steady between ages 17 and 37 years, increased less between ages 37 and 55 years, compared with females (5.2 vs. 7.9 mm Hg). Males and females had a similar increase in diastolic BP before age 17 years. Between ages 17 and 37 years, diastolic BP in men increased and then decreased slightly after 37 years, whereas in women, BP increased less steeply between ages 17 and 37 years and continued to increase after age 37 years.

Mean total cholesterol and fasting blood glucose levels were significantly different by sex at all 4 ages. Total cholesterol levels in females declined from ages 8 to 17 years (168 mg/dL to 163 mg/dL) and then increased at age 37 years and increased again to reach intermediate levels at age 55 years. Total cholesterol declined in males from ages 8 to 17 years (164–151 mg/dL) increased to 193 mg/dL at age 37 years, and decreased to 182 mg/dL at age 55 years. Total cholesterol levels were in males were lower than in females at all ages except 37 years. Blood glucose levels in males increased more than in females in every age period. Blood glucose levels in females consistently increased over time, from 80.7 mg/dL at age 8 years to 95.9 mg/dL at age 55 years.

In a sensitivity analysis including 14,422 individuals with complete parental education data, results were similar between the models with and without adjustment for parental education (16.2 years (95% CI: 15.5, 16.8) and 39.8 years (35.8, 43.8) without adjustment for parental education compared with 16.1 years (95% CI: 15.4, 16.7) and 39.3 years (35.5, 43.0) with adjustment).

## DISCUSSION

Within this pooled cohort, there were influential periods in adolescence and adulthood when the loss of clinical CVH was accelerated. The largest acceleration in the age-related loss of clinical CVH occurred at the first change point, at approximately age 17 years. A second, less dramatic acceleration in the age-related decline in clinical CVH occurred at the second change point, in middle age at age 37 years. These ages (approximately 17 and 37 years) appear to coincide with social and developmental transitions, providing unique insight to the development of preventive interventions tailored to these specific phases in life (18). CVH score slopes between change points as well as the patterns of change for the individual metrics comprising the score differed by sex, suggesting the potential importance of sex-specific interventions.

At age 17 years, adolescents typically gain increased independence from their parents and transition from pediatric to adult health care practitioners. Many adolescents leave their provider’s practice between ages 15 and 22 years, mainly because of aging out or completely dropping out of primary care (19, 20). This poses challenges for intervention, due to interrupted continuity of care. Males tend to have a longer gap in care than females (21). Changes in schedules, school, and jobs may interfere with meeting guidelines for healthy sleep, physical activity, and diet (22). There is also a large shift in personal responsibility, greater financial independence, with access to tobacco, greater personal choices around food selection, alcohol use, and physical activity at this time. These behaviors are important for achieving and maintaining ideal CVH (2). We observed low mean scores for diet among persons aged 17 years, suggesting diet may be an important lifestyle behavior to potentially target for intervention.

At approximately age 37 years, adults transition from young adulthood to middle age. The increasing personal, professional, and social pressures encountered during this phase of life may additionally compete with adherence to a healthy lifestyle, leading to diminished levels of clinical risk factors. Employment-based wellness programs could help facilitate maintenance of CVH during this period.

Although the second change point at age 37 years was slight in the overall model, there was a significant difference by sex, illustrating changes men and women experience over time. Our results show that although women maintained a higher CVH score through age 37 years, their rate of decline was significantly greater than that of men from ages 37 to 55 years. These results support well-documented data showing that men and women differ in their trajectories regarding CVH risk factor development over time (22). Although much remains unknown about CVH during and after pregnancy in women, pregnant women between the ages of 20 and 44 years are significantly less likely to maintain ideal CVH than are nonpregnant women in the same age range (23). The second change point falls squarely during women’s reproductive years and could be a contributing factor in the difference between men and women during this time. In addition to the difference in CVH decline, there are also differences in health care use by sex (24, 25). Men are less likely than women to use health care services or make preventive care visits despite declining health comparable to that of women (25).

Our findings on the age-related changes of individual CVH metrics complement prior work. Specifically, in most cross-sectional and cohort studies, researchers reported that BMI increases with age, possibly leveling out in older age (26–32). Although trends in BMI over time in this study did not significantly differ by sex, other authors have suggested, and we concur, that, ideally, intervention should differ for men and women (26). For example, Li et al. (33) concluded that high or increasing BMI at any life stage is associated with a high adult BP. Although BP increased over time in our data, it remained, on average, higher in men than women at most points after age 8 years (33). These 7 metrics are synergistic in children and young adults with comorbidities (34). Risk behaviors are interrelated—intervention in one can affect another (22).

We know that risk of cardiovascular disease is established long before middle age (35), and with primary and primordial prevention, cardiovascular disease can be prevented (36). The World Health Organization strongly recommends primordial and primary prevention of major known risk factors for noncommunicable diseases such as cardiovascular disease to begin early in life (37). Longitudinal analyses of CVH have shown that maintaining CVH from childhood onward reduces the risk for cardiovascular disease in adulthood (4, 5).

Our approach to determining influential age windows when loss of CVH accelerates has several strengths, including the large data set with multiple observations for individuals from childhood through middle age. The method for determining change points accounts for repeated measures within individuals, and we used a likelihood-based approach that does not require any prior assumptions (14). In 2 of the 5 cohorts in this pooled cohort analysis, all cohort members were White; therefore, a limitation of our work is that we were unable to estimate differences in change points or slopes by race without essentially performing cohort-specific analyses. Although we attempted to deal with cohort-specific differences by adjusting all models by cohort, there have been changes over time in standards of care and of public health messaging that may differ between and within the United States and Finland over time. Studies will be needed to examine how changes in clinical, behavioral, and cultural factors may affect these influential periods. Patterns of change in CVH may differ within a population. However, in multiple studies on CVH trajectories, a decline in CVH has been reported to occur during childhood and early adolescence, and there appears to be a change point near age 17 years for all trajectory groups (4, 5); these findings support the population-level influential age windows we estimated in the present study. Reviewing the means in each metric by age and sex, we were not able to take medication use into account, as is done in the CVH score. So trends in BP and levels of cholesterol and blood glucose may appear to be attenuated because of medications used to control these factors. In addition, data on CVH behaviors are sparse, particularly for younger ages, so we were not able to estimate change points in the full CVH score including these observations. Our data begin at age 8 years; therefore, we cannot generalize from these data how and when changes in overall CVH may occur in children younger than 8 years, but this should a topic of future investigation. Although we did estimate change points separately by sex, we did not have menopausal status available to us from every cohort; therefore, these biological changes were not included in our analysis.

In conclusion, adolescence and young adulthood are key age windows when age-related loss of clinical CVH greatly accelerates. Trends in CVH are not consistent by sex, and identification of specific age-windows offers an opportunity for personalized preventive intervention. Additional research identifying the specific factors leading to accelerated loss of CVH in adolescence and middle adulthood is needed. The creation of personalized interventions targeted to these critical age windows may offer new tools to preserve ideal CVH throughout the life course.

## Supplementary Material

Web_Material_kwab149
