# Supplementary material for: Influential Periods in Longitudinal Clinical Cardiovascular Health Scores
Source: Am J Epidemiol. 2021 May 20;190(11):2384–94. doi: 10.1093/aje/kwab149 (PMC8561125; doi:10.1093/aje/kwab149)

Influential Periods in Longitudinal Clinical Cardiovascular Health Scores

Amy E. Krefman, Darwin Labarthe, Philip Greenland, Lindsay Pool, Liliana Aguayo, Markus Juonala, Mika Kähönen, Terho Lehtimäki, R. Sue Day, Lydia Bazzano, Vito M.R. Muggeo, Linda Van Horn, Lei Liu, Larry S. Webber, Katja Pahkala, Tomi T. Laitinen, Olli Raitakari, Donald M Lloyd-Jones, and Norrina B. Allen

Contents:

Web Table 1. Behavioral cardiovascular health (CVH) score components.

Web Table 2. Demographics, clinical measures, and covariates at each individual's first exam, by age.

Web Table 3. Model fit statistics.

Web Table 4. Change point estimates, by sex and race-sex strata.

Web Table 5. Mean values of each ideal behavioral score, by sex.

Web Figure 1. Sample Flow Diagram.

Web Figure 2. Estimated change points and their 95% confidence intervals, by sex and race-sex strata.

Web Figure 3. Boxplots of clinical measures – A) BMI, B) SBP, C) DBP, D) Total Cholesterol, and E) Fasting Glucose at ages 8, 17, 37, and 55 years, by sex.

Web Table 1. Behavioral cardiovascular health (CVH) score components.

	**Physical Activity**		**Diet**		**Smoking**	
**Goal/Metric**	<20 years of age	20+ years of age	<20 years of age	20+ years of age	<20 years of age	20+ years of age
**Ideal Health**	≥60 min of moderate- or vigorous-intensity activity every day	150+ minutes of moderate exercise or 75+ minutes of vigorous exercise/week	4-5 components of healthy diet	4-5 components of healthy diet	Never tried; never smoked whole cigarette	Never smoker or quit >12 months ago
**Intermediate Health**	>0 and <60 min of moderate or vigorous activity every day	1-149 minutes of moderate exercise or 1-74 minutes of vigorous exercise/week	2-3 components of healthy diet	2-3 components of healthy diet		Former smoker, quit ≤12 months ago
**Poor Health**	No exercise	No exercise	0-1 components of healthy diet	0-1 components of healthy diet	Tried prior 30 days	Current smoker

Web Table 2. Demographics, clinical measures, and covariates at each individual's first exam[a], by age.

Characteristics	8 to <17 years N=10,218	17 to <37 years N=11,924	37 to 55 years N=7,379
**Female,** No. (%)	5062 (49.54)	6475 (54.30)	4073 (55.20)
**White,** No. (%)	7251 (70.96)	7900 (66.25)	4960 (67.22)
**Age,** Mean (SD)	11.58 (2.46)	23.83 (4.88)	39.76 (2.64)
**Cohort,** No. (%)			
Young Finns	1350 (13.21)	2590 (21.72)	1941 (26.30)
Project HeartBeat!	407 (3.98)	40 (0.34)	0 (0.00)
CARDIA	0 (0.00)	5075 (42.56)	4192 (56.81)
Bogalusa	8064 (78.92)	3794 (31.82)	1246 (16.89)
STRIP	397 (3.89)	425 (3.56)	0 (0.00)
**Clinical measures,** Mean (SD)			
BMI, kg/m^2	19.08 (3.97)	24.29 (5.23)	28.13 (6.53)
BMI %, by age and sex	53.86 (30.06)	49.95 (29.71)	. (.)
SBP, mmHg	103.57 (10.84)	112.89 (11.72)	115.53 (14.75)
SBP %	49.26 (26.93)	47.43 (27.91)	. (.)
DBP, mmHg	51.99 (12.34)	67.01 (10.28)	74.23 (11.02)
DBP **%**	26.88 (23.39)	30.20 (24.16)	. (.)
Total **c**holesterol, mg/dL	165.44 (30.20)	176.83 (35.57)	190.14 (37.38)
Fasting glucose, mg/dL	84.55 (10.78)	83.98 (15.28)	94.40 (22.20)
**Ideal behavior scores,** No. (%)[b]			
Smoking	5770 (74.70)	5703 (51.45)	3228 (46.69)
Diet	357 (16.00)	2667 (31.84)	1052 (45.23)
Physical **a**ctivity	303 (15.33)	2419 (29.42)	1798 (28.24)
**Maternal education,** No. (%)[c]			
≤ 6 years	156 (2.20)	436 (4.48)	393 (5.70)
>6-9 years	1105 (15.57)	1552 (15.95)	1118 (16.23)
>9-12 years	3152 (44.43)	4300 (44.19)	2914 (42.30)
>12-16 years	2378 (33.52)	2605 (26.77)	1780 (25.84)
>16 years (grad school)	304 (4.28)	837 (8.60)	684 (9.93)
**Paternal education,** No. (%)[c]			
≤ 6 years	178 (3.01)	542 (6.01)	462 (7.14)
>6-9 years	988 (16.71)	1799 (19.96)	1373 (21.21)
>9-12 years	2621 (44.33)	3666 (40.67)	2442 (37.72)
>12-16 years	1868 (31.59)	2174 (24.12)	1498 (23.14)
>16 years (grad school)	258 (4.36)	833 (9.24)	699 (10.80)

**Clinical CVH Score**, Mean (SD)	7.13 (1.07)	6.67 (1.34)	5.54 (1.73)

Abbreviations: CARDIA, Coronary Artery Risk Development in Young Adults; STRIP, Special Turku Coronary Risk Factor Intervention Project; BMI, body mass index; SBP, systolic blood pressure; DBP, diastolic blood pressure; CVH, cardiovascular health; SD, standard deviation

[a] These are longitudinal data, the first observation in each window was included per participant, but individuals may contribute observations in more than one age window. [b] Behavior scores were not captured at every visit for every participant. [c]Parent education data were not available for all individuals.

Web Table 3. Model fit statistics.[a]

Model	Model #	Degrees of Freedom	AIC	BIC	Test	Likelihood Ratio	p-value
Linear, no change points	1	12	175006.8	175114.2			
Quadratic, no change points	2	13	174324.5	174440.8	1 vs 2	684.3455	<.0001
Cubic, no change points	3	14	174091.0	174216.3	2 vs 3	235.4926	<.0001
Linear, 1 change point	4	13	173980.0	174096.3	3 vs 4	108.9937	<.0001
Linear, 2 change points	5	14	173952.8	174078.0	4 vs 5	29.2277	<.0001

[a] all models are adjusted for race, sex, and cohort and include a random intercept and random slope.

Web Table 4. Change point estimates, by sex and race-sex strata.

Model	$N_{persons}$	$N_{observations}$	Change Point 1	Change Point 2
Male	8883	26189	16.93 (16.25, 17.61)	35.69 (33.09, 38.29)
Female	9461	30650	16.71 (15.94, 17.47)	36.06 (34.62, 37.49)
White Female	6288	19792	15.82 (15.13, 16.52)	38.33 (36.46, 40.19)
Non-white female	3173	10858	17.41 (15.45, 19.37)	32.92 (29.66, 36.18)
White male	6059	17711	15.85 (15.12, 16.57)	40.87 (36.76, 44.98)
Non-white male	2824	8478	18.77 (17.34, 20.2)	37.00 (33.65, 40.34)

Web Table 5. Mean values of each ideal behavioral score, by sex.

**Age and Sex**	**Mean(SD) or Difference(95% CI) in CVH Score: Poor(0) to Ideal (2)**					
	**Diet**		**Physical Activity**		**Smoking**	
	N	Mean (μ)	N	Mean (μ)	N	Mean (μ)
**8-year-olds**[a]						
**Female**	236	1.1(0.7)	224	0.9(0.4)	534	1.5(0.9)
**Male**	214	1.0(0.7)	210	0.9(0.4)	483	1.5(0.9)
**Difference in mean (μ_{Male}- μ_{Female})**		-0.1(-0.2-0.02)		0.02(-0.04 – 0.1)		-0.02(-0.1- 0.1)
**17-year-olds**						
**Female**	216	0.5(0.6)	198	1.2(0.4)	659	1.3(1.0)
**Male**	213	0.3(0.5)	194	1.3(0.5)	628	1.2(1.0)
**Difference in mean (μ_{Male}- μ_{Female})**		**-0.2(-0.3 - -0.1)***		0.1(-0.02 – 0.2)		**-0.1(-0.2 - -0.0)***
**37-year-olds**						
**Female**	226	1.6(0.6)	710	1.0(0.7)	785	1.1(0.9)
**Male**	168	1.5(0.7)	564	1.3(0.7)	631	1.1(0.9)
**Difference in mean (μ_{Male}- μ_{Female})**		-0.1(-0.2 – 0.1)		**0.3(0.2 – 0.3)***		-0.1(-0.2 – 0.03)
**55-year-olds**[b]						
**Female**	219	1.9(0.3)	186	1.1(0.8)	185	1.8(0.6)
**Male**	150	1.8(0.4)	131	1.3(0.7)	129	1.8(0.6)
**Difference in mean (μ_{Male}- μ_{Female})**		-0.06(-0.1-0.01)		**0.2(0.1-0.4)***		0.0(-0.1-0.2)

Abbreviations: SD, standard deviation; *Indicates P<0.05 for t-test

[a]Diet and Physical Activity data are from 8-9 year olds due to lack of data at age 8 (n=1 for both metrics).

[b]Diet data are from 50-55 years due to lack of diet data at age 55 (n=2).

Web Figure 1. Sample Flow Diagram

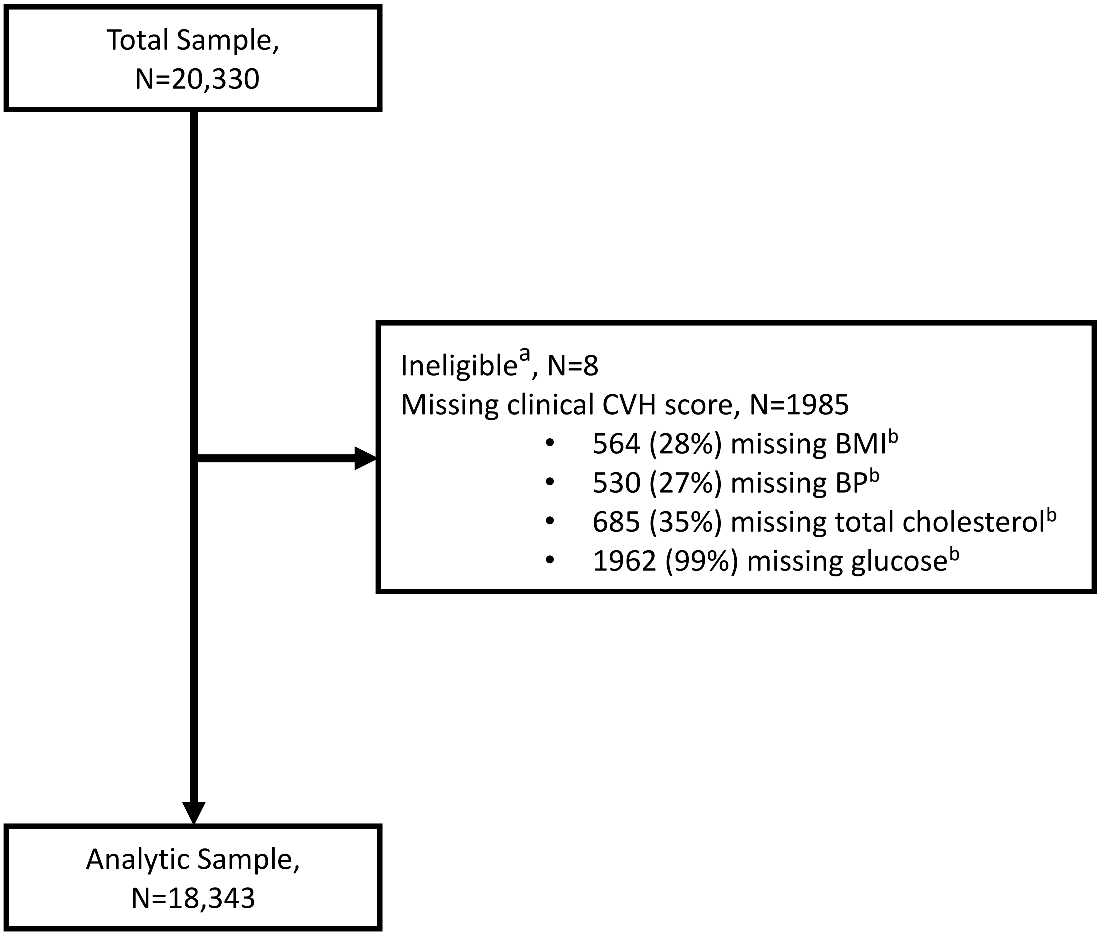

[a] Ineligible due to removal from analysis by cohort, or all observations outside the age range 8-55 years.

[b] Missing metrics are not mutually exclusive.

Web Figure 2. Estimated change points and their 95% confidence intervals, by sex and race-sex strata.

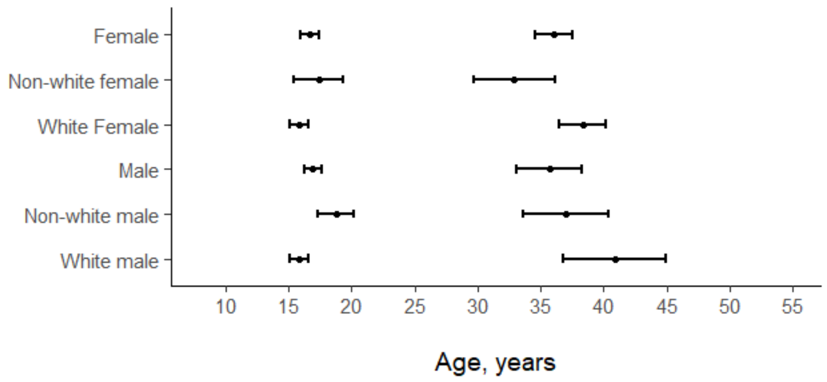

Web Figure 3. Boxplots of clinical measures – A) BMI, B) SBP, C) DBP, D) Total Cholesterol, and E) Fasting Glucose at ages 8, 17, 37, and 55 years, by sex.

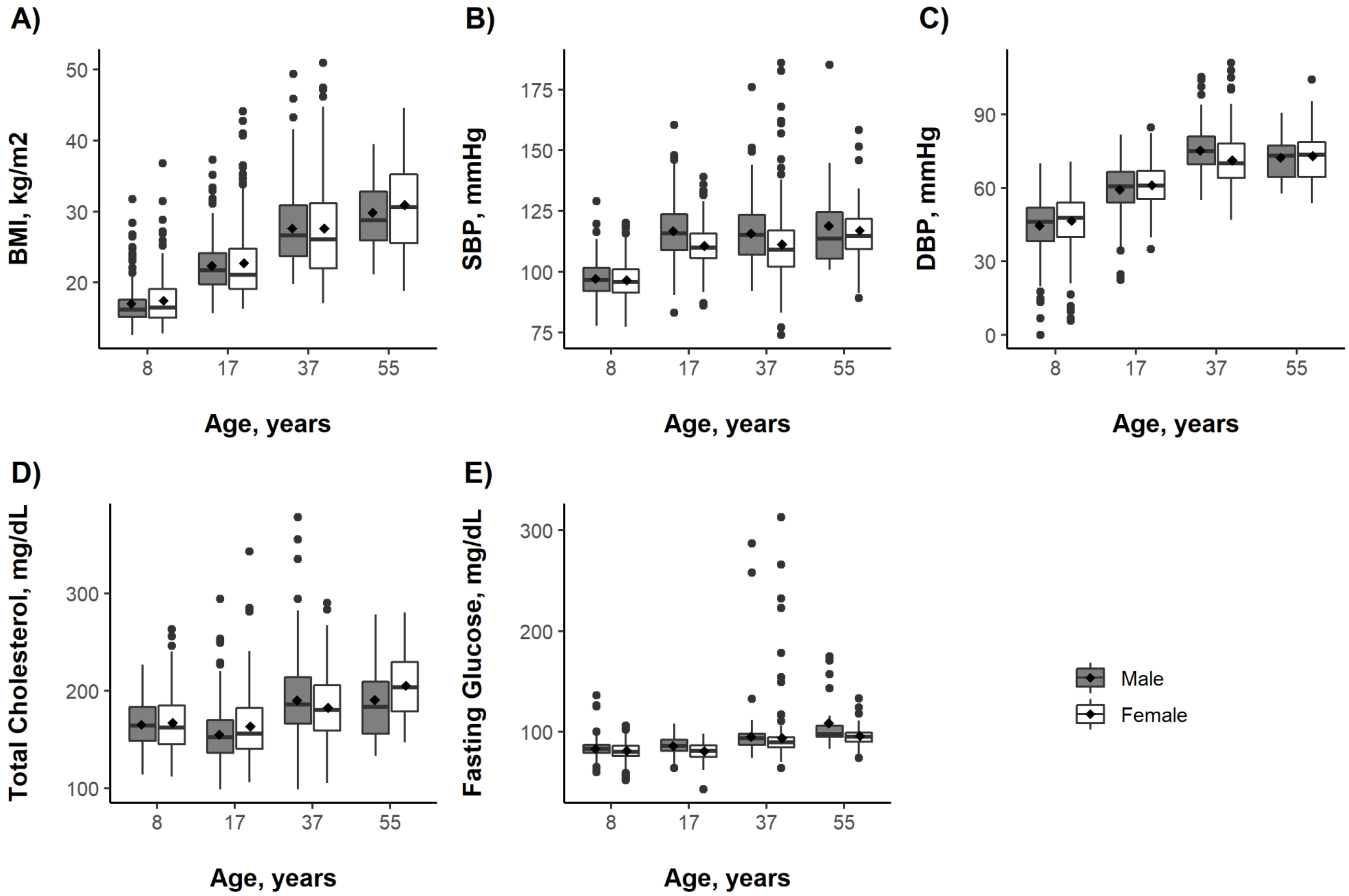

Supplement: Web_Material_kwab149 [file web_material_kwab149.pdf]
